# The MindfulBreather: Motion Guided Mindfulness

**DOI:** 10.3389/fnhum.2017.00613

**Published:** 2017-12-15

**Authors:** Tom B. Mole, Julieta Galante, Iona C. Walker, Anna F. Dawson, Laura A. Hannah, Pieter Mackeith, Mark Ainslie, Peter B. Jones

**Affiliations:** ^1^Department of Psychiatry, University of Cambridge, Cambridge, United Kingdom; ^2^Department of Psychology, University of Glasgow, Glasgow, United Kingdom; ^3^Faculty of Education, University of Cambridge, Cambridge, United Kingdom; ^4^Cambridgeshire & Peterborough NHS Trust, Cambridge, United Kingdom; ^5^Faculty of Medicine and Health Sciences, University of East Anglia, Norwich, United Kingdom; ^6^Heart Institute, University of Ottowa, Ottawa, ON, Canada

**Keywords:** meditation, mindfulness training, default mode, realtime, MindfulBreather, motion guided mindfulness, psychobiological synchronization model, mindfulness assessment

## Abstract

For millennia, humans have focused their attention on the breath to develop mindfulness, but finding a scientific way to harness mindful breathing has proven elusive. Existing attempts to objectively measure and feedback on mindfulness have relied on specialist external hardware including electroencephalograms or respirometers that have been impractical for the majority of people learning to meditate. Consequently, training in the key skill of breath-awareness has lacked practical objective measures and guidance to enhance training. Here, we provide a brief technology report on an invention, The MindfulBreather^®^ that addresses these issues. The technology is available to download embedded in a smartphone app that targets, measures and feedbacks on mindfulness of breathing in realtime to enhance training. The current article outlines only the technological concept with future studies quantifying efficacy, validity and reliability to be reported elsewhere. The MindfulBreather works by generating Motion Guided Mindfulness through interacting gyroscopic and touchscreen sensors in a three phase process: Mindfulness Induction (Phase I) gives standardized instruction to users to place their smartphone on their abdomen, breathe mindfully and to tap only at the peak of their inhalation. The smartphone’s gyroscope detects periodic tilts during breathing to generate sinusoidal waveforms. Waveform-tap patterns are analyzed to determine whether the user is mindfully tapping only at the correct phase of the breathing cycle, indicating psychobiological synchronization. Mindfulness Maintenance (Phase II) provides reinforcing pleasant feedback sounds each time a breath is mindfully tapped at the right time, and the App records a mindful breath. Lastly, data-driven Insights are fed back to the user (Phase III), including the number of mindful breaths tapped and breathing rate reductions associated with parasympathetic engagement during meditation. The new MGM technology is then evaluated and contrasted with traditional mindfulness approaches and a novel Psychobiological Synchronization Model is proposed. In summary, unlike existing technology, the MindfulBreather requires no external hardware and repurposes regular smartphones to deliver app-embedded Motion-Guided Mindfulness. Technological applications include reducing mindwandering and down-regulation of the brain’s default mode through enhanced mindful awareness. By objectively harnessing breath awareness, The MindfulBreather aims to realize the ancient human endeavor of mindfulness for the 21st century.

## Introduction

For millennia, humans have focused their attention on the breath to develop mindfulness, but finding a scientific way to harness mindful breathing has proven elusive. Attempts to objectively link mindfulness using external hardware such as respirometers (Levinson et al., 2014), EEG (Bhayee et al., 2016) or realtime fMRI machines (Garrison et al., 2013) have proven too cumbersome, expensive and inaccessible to be used by most people. Whilst there are hundreds of mindfulness app currently available (Mani et al., 2015), none are able to provide inbuilt objective and realtime measurement or guidance to build breath-awareness. Consequently, there is a need to develop a simple and scalable mindful breathing technology that doesn’t need impractical external devices and can work within an App for widespread use.

There is a strong rationale for developing objective measures and realtime training for mindfulness for several reasons. First, there have been calls for developing objective ways of measuring mindfulness because subjective self-report questionnaires of mindfulness suffer from several limitations (Sauer et al., 2013). These limitations include several biases including those related to recall, practice effects and social desirability (Baer, 2011; Bergomi et al., 2013). Second, realtime feedback from objective measurement has been highlighted for its potential to help drive plasticity in brain function, cognition and accelerate learning to improve a range of mental health outcomes (Stoeckel et al., 2014). Hence there is a need to provide objective realtime feedback technology to enhance the measurement and learning of mindfulness techniques that are commonly used to improve wellbeing.

Here, we outline an invention, The MindfulBreather^®^ (International Patent Application Publication No. WO 2016/181148 A2; (Mole, 2015) Mindz Training LTD, Cambridge, UK) that overcomes these issues by creating App-embedded Motion Guided Mindfulness. A free version will be made available for public download from August 2017, alongside training materials within the “Mindz” App^1^. This technology objectively induces, regulates and provides insights into mindful breath-awareness through an innovative smartphone-embedded gyroscopic-guidance system. Because of the ubiquity of inbuilt gyroscopes in smartphones, The MindfulBreather App could be made accessible to anyone with just a modern mobile phone.

## Materials and Methods

**Figure 1:**
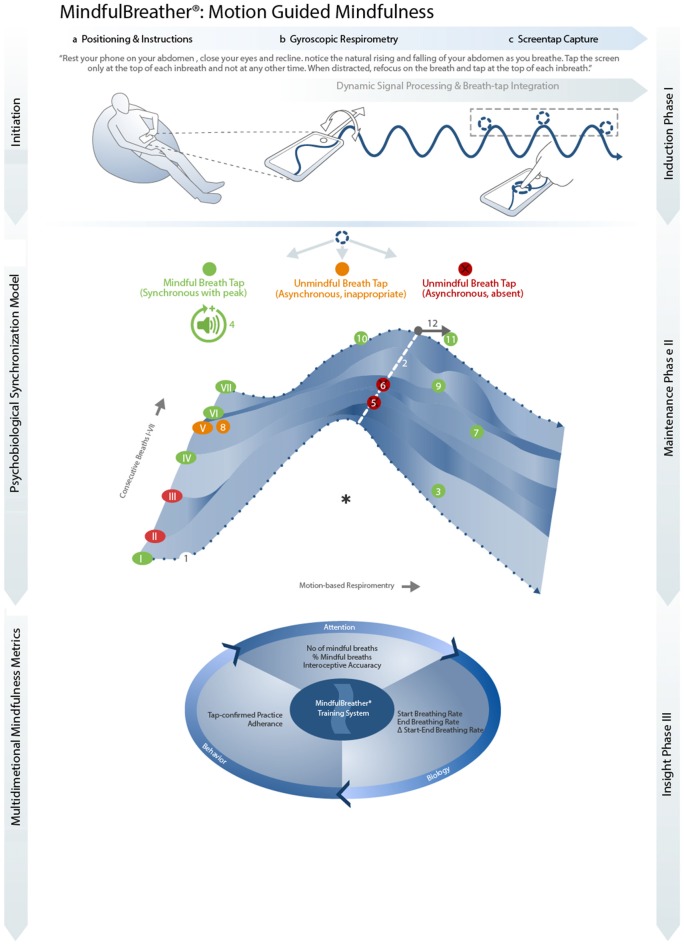
*Illustrative 7-breath Meditation| (1) Gyroscopic Respirometry Trace. As the user breaths in, the gyroscopic trace rises, as the user breaths out the trace falls to create peaks. In this illustration, waveform peaks are extracted from the continuous waveform and concatenated to show the first seven consecutive inbreath peaks (I–VII; green = mindfulbreaths, orange and red = unmindful breaths). (2) Target Tap-time: the Peak of each inbreath is analyzed and established as the target time when users are expected to tap if mindful (white dotted line). (3) Mindful Breath (Low-accuracy Tap): shortly after the first peak inhalation, as the user is aware of their breath, the user taps the screen. Because the tap is not far from the peak inhalation, the tap is classified as time-appropriate and the breath is classified as a mindful breath. (4) Positive Reinforcement: as the breath was classified as a mindful breath, a pleasant sound is triggered to give user feedback and reinforce breath-awareness. (5,6) Unmindful Breaths (missed taps) The user then gets distracted with mindwandering so continues to breathe but does not tap at the peak of their next two inhalations due to inattention. These two breaths are classified as unmindful. (7) Refocused Mindful Breath. The user then notices they are distracted and refocuses awareness in time to mindfully tap the next breath shortly after the peak inhalation. (8) Unmindful Breath (time-inappropriate tap). The user then gets distracted again and this time guesses when to tap. Because this tap is too early and far from the peak, this is classified as an unmindful breath. (9–11) Mindful Breath (Higher-accuracy Tap). The user refocuses attention before the next breath to tap close to the peak on three consecutive breaths (9–11) indicating higher breath awareness, particularly for the final breath (11), showing accurate momentary awareness (12).

The MindfulBreather works by generating *Motion Guided Mindfulness* through interacting gyroscopic and touchscreen sensors in a three phase process (see Figure 1):

### Phase I: Mindfulness Induction

First, appropriate positioning and mindfulness induction is achieved with standard instructions inviting the user to recline, position their smartphone on their abdomen and pay mindful attention to their breath (Figure 1; Panel 1a). Second, G*yroscopic Respirometry* is performed (Figure 1; Panel 1b). With the user’s phone resting on their abdomen, the abdominal cavity naturally expands and recoils during the respiratory cycle causing the smartphone to periodically tilt. Resulting pitch fluctuations are detected by the phone’s inbuilt gyroscope/accelerometer sensors. Algorithmic analysis of the sinusoidal peaks and troughs produced, indicates the current phase of the breath, after smoothing and signal processing techniques are applied. Third, *Screen Tap Capture* is achieved by recording each time the user touches the screen (denoted by circles alongside the breathing waveform in Figure 1; Panel 1c) relative to a specific biological phase of breathing—the peak of the inhalation.

### Phase II: Mindfulness Maintenance

User tapping data are analyzed and overlaid on respiratory waveform data to generate an integrated *Psychobiological Synchronization* *Model* of mindful breathing (Figure 1; Panel 2). Moment-by-moment, each breath is analyzed and categorized according to the tap pattern detected. A mindful breath is determined by a screen-tap that is near the inhalation peak, time-appropriate and hence synchronized with the biological process of breathing (depicted here by green circles). An unmindful breath is demonstrated by a screen-tap that is absent (depicted by red circles) or time-inappropriate (depicted by orange circles) and hence asynchronous with the inhalation peak. Only after a mindful breath, is a pleasant sound triggered for immediate positive reinforcement to provide maintenance of the state of mindfulness. Maintenance of awareness is further maintained through integrated gamification and Hawthorne learning effects, as users are made aware of each mindful breath as it is digitally recorded. Further features of the model are explained through the illustrative 7-breath meditation in Figure 1 and accompanying caption. Overall, *Categorical Mindful Inference* first classifies whether the breath is mindful or unmindful depending on tapping patterns. Subsequently, *Quantitative Mindful Inference* measures the proximity of taps compared to the inhalation peaks to derive interoceptive accuracy, a key metric that has been associated with meditation practice (Khalsa et al., 2008). In addition, *Concurrent Mindful Inferences* can be made by monitoring variables strongly associated with mindfulness: reduction in breathing rate during meditation assesses enhanced vagal tone consistent with the physiological relaxation response expected during meditation. The duration of time spent practicing whilst tapping appropriately can objectively assess behavioral adherence to mindfulness practice.

### Phase III: Mindfulness Insights

Finally, multidimensional mindfulness metrics can be fed back to the practitioner to offer understanding on distinct components of mindfulness practice. Longitudinal tapping and respiratory rate trends can be compared after several weeks of training to assess for practice-dependent neuroplasticity. This feedback enhances self-awareness and allows for assessment of the users response to their training whilst also providing individualized insights into mindfulness metrics.

## Discussion

In summary, the invention of the MindfulBreather^®^ technology exploits Motion Guided Mindfulness to scientifically harness mindfulness of breathing. Unlike existing technology, The MindfulBreather does not rely on subjective self-report (Frewen et al., 2016), and requires no specialist hardware beyond a widely-available, regular smartphone. This MGM training paradigm not only offers unprecedented parsimony, portability and scalability but contrasts with traditional mindfulness training in several important respects (see Table 1).

**Table 1:** Differentiating mindfulness training methods.

Aspect	Traditional training	MindfulBreather training
Measurement	Subjective	Objective
Psychological Therapeutic Targeting	Implicit	Explicit: Mindful Breathing demonstrated by interoceptive task-positive upregulation and consequent task-negative “default mode” downregulation
Biological Therapeutic Targeting	Implicit	Explicit: Enhanced parasympathetic activity as measured by reductions in breathing rate during meditation
Theoretical Model Employed	Implicit/Variable	Explicit: Psychobiological Synchronization Model
Metrics Yielded	Commonly Unidimensional: Psychological (typically questionnaires only);	Multidimensional: attentional; physiological; behavioral
Training Delivery; Format; Integration of Multimedia-based Learning	Face-to-face; didactic; No	App-embedded smartphone delivery; Interactive Motion-Guided Mindfulness; Yes
Learning Reward	Implicit	Explicit: Operant conditioning positively reinforces breath-awareness with immediate pleasant auditory feedback sounds
Mindfulness Assessment and Intervention Processes	Separated	Integrated
Feedback Temporality and Temporal Resolution; Ecological Validity	Periodic/static; low resolution; low	Realtime/dynamic; high resolution; high (users can practice in natural settings)
Mindfulness Training enhanced with Digital Hawthorne Effect and Gamification-Based Learning	No; No	Yes; Yes
Adherence Monitoring	Analog and Subjective: subject to recall and self-report biases	Digital and Objective: confirmed by response and tapping patterns consistent with ongoing mindfulness practice
Accessibility, Affordability and Portability	Low	High
Scalability	Low	High

Most importantly, traditional methods of learning mindfulness offer no objective feedback. The main advantage of this novel approach is that it provides objective real-time feedback, that could accelerate learning of self-regulation (Stoeckel et al., 2014). There are also other specific advantages worthy of note: first, users are rewarded when engaging appropriately in the task by hearing a reassuring bell each time they tap a mindful breath. Second users receive tangible and objective results of how many mindful breaths they have taken that substantiate and justify their cognitive effort required during meditation. Third, the technology demonstrates to users the degree to which they have activated the relaxation response by measuring the change in breathing rate during meditation. This in turn supports learning through feedback of a wide range of different metrics to support self-insight and self-regulation.

With this technology, objective attentional, physiological and behavioral mindfulness-related measures are captured automatically providing a more comprehensive, triangulated assessment of metrics related to mindfulness. This technology further offers a novel psychobiological synchronization model for understanding and regulating mindful breathing. A limitation of the system is that the user cannot sit upright or the phone will lose contact with the abdomen, though the reclined position (ranging from recumbent to sitting almost fully upright—a range of hip flexion of approximately 0–70°) allows sustained practice without fatiguing postural muscles or causing discomfort for novice meditators. From the perspective of a 2-component definition of mindfulness comprising attention and acceptance (Bishop et al., 2004), the technology objectively measures and trains the former but subjectively regulates the latter though the standardized instructional script.

Breath-awareness indicated by the timely tapping of a specialized digital device has been previously systematically validated in a multi-study approach as a reliable measure of mindfulness that is sensitive to change after training (Levinson et al., 2014). Notably, this approach does introduce a small secondary task of periodic device-tapping. However this is unlikely to detract significantly from the primary interoceptive task for three main reasons. First, interaction with the device is momentary and takes a very small amount of time proportionally compared to the act of passively observing the breath without any motor activity. Second, the motor activity required to button-press is minimal and recruits small number of muscles in the hand in a familiar action. Third, the cognitive complexity of this action is minimal and so such as simple action is likely to have minimal effects on cognition or memory and would not require significant motor planning. Taken together, using a brief, negligibly complex motor task in parallel with breath-awareness appears to be both a reliable and valid way of teaching mindful breathing.

By responding and reinforcing individuals’ attentional control in realtime, this approach supports other developments aimed at personalizing mindfulness training (Van Dam et al., 2015). Through measuring mindful breaths, the technology could be applied to create a novel standardized “currency” for quantifying practice intensity and duration. The App also has potential application as an instrument for modulating brain processing modes from a task-negative, default mode to a task-positive interoception mode. As such, it holds promising potential as an effective instrument that targets key mechanisms implicated in meditation including disengagement of mindwandering (Brewer et al., 2011) and subserving brain networks and more broadly as an enhancer of attentional regulation and self-regulation (Tang et al., 2015). Taken together, the technology transforms and repurposes a regular smartphone into a motion guided mindfulness training environment enhanced by multidimensional momentary metrics. Pilot studies are planned to clinically validate and refine this instrument across different age groups and phenotypes, both as a measurement device and a training device. We hypothesize stronger, objective biomarkers and guidance during meditation training will enhance outcomes. By progressing beyond subjective breath awareness and harnessing previously elusive objectivity, The MindfulBreather aims to advance the ancient human endeavor of mindfulness and its pragmatic realization in the 21st century.

## Author Contributions

TBM conceived the article and all authors helped design the work and critically revised the article for intellectual content. All authors reviewed and approved the final manuscript.

## Conflict of Interest Statement

The authors JG, ICW, AFD, PM, LAH, MA and PBJ declare no competing financial interests and received no commercial funding. Author TBM has been awarded a place by The Cambridge Centre for Social Innovation, Cambridge Social Ventures Incubator and is director and shareholder of Mindz Training LTD that developed the MindfulBreather^®^, International Patent Publication No. WO 2016/181148 A2.

## Footnotes

^1^http://www.mindz.com
